# A comparison of the upper anterior teeth movements with optimized and conventional attachment

**DOI:** 10.4317/jced.61466

**Published:** 2024-04-01

**Authors:** Tahara Hassanaly, Ana Rabal-Solans, María-Carmen Mediero-Pérez, Iván Nieto-Sánchez

**Affiliations:** 1 DDS, MSc. Postgraduate Student, Alfonso X el Sabio University, Madrid, Spain; 2 DDS, MSc. Private Practice, Madrid, Spain; 3 DDS, MSc, PhD. Assistant Professor, University Complutense, Madrid, Spain; 4 DDS, MSc, PhD. Hospital Universitario San Rafael-Francisco de Vitoria University

## Abstract

**Background:**

Invisalign® attachments are divided into two main groups: the conventional group and the optimized group, which is also known as the SmartForce™. The aim of this study is to compare the movements produced by an optimized crescent-shaped attachment in superior incisor teeth with the movements produced by a conventional rectangular attachment (vertical and horizontal) in the same teeth.

**Material and Methods:**

This retrospective study examined the movement table of the initial ClinCheck® and the first refinement of 95 patients (mean age 44.18 ± 4.125, 40 males and 55 females). It represented 147 upper incisors divided into two groups: 87 with rectangular attachment and 60 with optimized attachment. Applying Kravitz’s accuracy formula for each movement and each tooth of interest (with attachments of interest), we underlined the effectiveness of each attachment. Mann–Whitney U test, Kruskal–Wallis test, and Pearson and Spearman correlation coefficients were used for statistical analysis.

**Results:**

The accuracies of rotation, mesio-distal angulation and vestibulo-lingual inclination are highly significantly related to the type of attachment used on the upper lateral incisor. The optimized attachment presented greater accuracy in the rotation of the lateral incisors than the conventional attachment. However, conventional vertical attachment showed a higher accuracy (*p*<.01) in the mesio-distal angulation and horizontal attachments showed a higher accuracy (*p*<.01) in the vestibulo-lingual inclination in the same group of teeth.

**Conclusions:**

Optimized attachments rotate better lateral incisors; conventional vertical attachments are more efficient to improve mesio-distal angulation; and horizontal attachments serve best for torque movements.

** Key words:**Orthodontics, clear aligners, orthodontic tooth movements, attachments.

## Introduction

Since Chishti and Wirth (1) introduced Invisalign® in 1998, there has been a considerable increase in demand for and development of invisible orthodontics. Indeed, in the last few years, aggressive marketing policies and advertisements in the social media have put clear aligners (CA) in the public spotlight (2). Nowadays, Invisalign® continues to be at the forefront of CA treatment (3).

Those aligners are manufactured with a semi-elastic transparent polyurethane material (4) (5). Initially, the aligner was programmed to move the tooth in the range of 0.25–0.33 mm in 14 days. However, in 2016, Invisalign® replaced its protocol, changing the aligner every seven days while keeping the same range of tooth movement (6)(7).

Attachment design is a fundamental aspect of the diagnosis and treatment planning because it helps in the correct retention of the CA, supports complex movements, and increases the accuracy to achieve the movement (8,9).

To achieve a specific movement, the orthodontist can choose between an automatically placed optimized attachment (which is part of the SmartForce™) or a conventional one (ellipsoid, rectangular or beveled rectangular).There are as many optimized attachments as there are dental movements (e.g. rotation, extrusion/intrusion, tip) (10). For instance, the software will automatically place an extrusion optimized attachment in the incisors if the threshold is superior at 0.5 mm (correcting 0.25 mm per stage) (11).

The aim of this study is to investigate which type of attachment allows the most precise tooth movement to simplify the daily clinical decision of the operator.

## Material and Methods

Ethics approval was obtained from the Ethics Committee of the research project of Alfonso X University on 02/07/2023 (protocol number 2023_02/158).

This research aims to test the following null hypothesis H0: “There are no differences between conventional and optimized attachments, and we achieved the same results with either attachment on upper incisors.”

This retrospective study examined the movement table of the initial ClinCheck® and the first refinement of 95 patients treated between 2018 and 2022. It represented 147 upper incisors divided into two groups: 87 with rectangular attachment and 60 with optimized attachment.

The sample size was estimated based on previously published data on the standard deviation for incisor root movements (SD 4.9 degrees) (12). By setting type I error at 0.05 and type II error at 0.10 (i.e. 90% power), it was estimated that 44 cases were sufficient to detect a clinically relevant difference in root movement of ≥5 degrees.

Patients were selected according to the following inclusion criteria: patients from Alfonso X University and from a private orthodontic clinic who had completed their treatment and presented a refinement ClinCheck®; attachments of interest present on the initial ClinCheck®; patients with malposition in the upper incisors; both sexes; aged between 16 and 66 years; and patients who collaborate with compliance hours.

We divided the patients into six groups according to the upper incisor (1 for central and 2 for lateral) and according to the attachment (O for optimized, V for conventional vertical and H for conventional horizontal): 1O, 1V, 1H, 2O, 2V and 2H.

For each group, we measured seven types of movements: extrusion (+)/intrusion (−), relative extrusion (+)/intrusion (−), vestibular (+)/lingual (−) translation, mesial (+)/distal (−) translation, mesial (+)/distal (−) rotation, mesial (+)/distal (−) angulation and vestibular (+)/lingual (−) inclination.

The movement Table of the initial ClinCheck® is our prediction or the movement theoretically attainable. The movement Table of the first refinement is the achieved movement. To evaluate the accuracy of the movement, we used the Kravitz formula: 100 − [(|predicted-achieved|)/|predicted|] × 100.

We then compared, respectively, for the central and lateral upper incisors, the accuracy of optimized, vertical and horizontal attachments for the seven tooth movements.

-Statistical analysis

SPSS software (IBM Corp. Released 2021. IBM SPSS Statistics for Windows, Version 28.0. Armonk, NY: IBM Corp) was used. Kolmogorov–Smirnov test was used to check the normality of the sample. Mann–Whitney U test, Kruskal–Wallis test, and Pearson and Spearman correlation coefficients were used to analyse the movement efficiency of different aligners.

## Results

Table 1 summarizes the distribution of the groups. 2O has the greatest number of interventions (40.1%), followed by 2V (37.4%), 2H (14.3%), 1V (5.4%), 1H (2%) and 1O (0.7%).

**Table 1:**
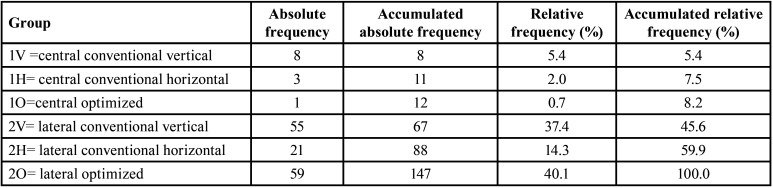
Distribution of the groups.

Therefore, all the highly significant values were found in the upper lateral incisor group.

Table 2 displays that the accuracy of mesio-distal rotation, mesio-distal angulation and vestibulo-lingual inclination is significantly and highly dependent on the attachment type in the upper lateral incisor.

**Table 2:**
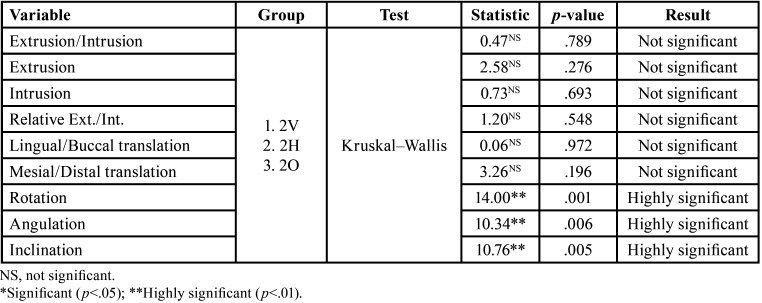
Accuracy of the different movements in upper lateral incisor according to the type of attachment.

Table 3 summarizes the following:

**Table 3:**
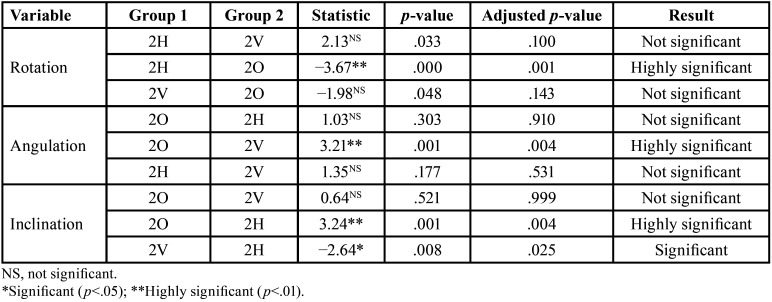
Accuracy of rotation, angulation and inclination in the upper lateral incisor according to the type of attachment.

1. Optimized attachment increases the rotation accuracy compared to horizontal attachment.

2. Vertical attachment increases the mesio-distal angulation accuracy compared to optimized attachment.

3. Horizontal attachment increases the vestibulo-lingual attachment accuracy compared to vertical and optimized attachments.

## Discussion

In his systematic review, Rossini *et al*. (13), emphasized that the less precise movement of Invisalign® for the upper incisors is extrusion followed by rotation. Likewise, Laohachaiaroon *et al*. (14) found that the extrusion accuracy of superior incisors was 18.3%. Indeed, superior lateral incisors have a reduced size that is difficult for tooth movement, especially for extrusion movement (15).

On the contrary, for intrusion, by measuring the anterior sector only to minimize bias, there is a high accuracy for the central (91.1%) and lateral (91.8%) incisors for 2 mm of intrusion maximum (16). Yet, Haouili *et al*. (17) highlighted that even with the G5 features (pressure areas and bite ramps) intrusion of incisors was still a challenge and did not improve since the study by Kravitz *et al*. (18) in 2009. Rotation movement is predicTable if we rely on Lombardo *et al*.’s study (19), which acknowledges that superior incisor rotation is more accurate than inferior premolars.

We rejected the null hypothesis H0 for specific movements (rotation, mesio-distal angulation and vestibulo-lingual inclination). We found that for this movement, the optimized attachment has greater accuracy. However, our results are not in accordance with those of Xie *et al*. (20) who underlined that the operator could maintain either the optimized attachment or the power ridge that is automatically put or dragged in a conventional attachment. Karras *et al*. (21) supports Xie’s finding. There are no sufficient clinical or statistical differences to demonstrate that one attachment has better accuracy in the rotation of the upper incisors.

Bates *et al*. (22) carried out a study that evaluated the effectiveness perceived by dentists and orthodontists, respectively, on the conventional and optimized attachment effects to extrude an upper lateral incisor. This cross-sectional survey study revealed that dentists were significantly more likely to use an optimized attachment as suggested by the ClinCheck® program, while orthodontists were significantly more likely to select a horizontal rectangular attachment that was beveled gingivally and created additional space around the tooth.

Savignano *et al*. (23) defined the rectangular attachment on the palatal side of the upper central incisor as the conFiguration to extrude this tooth. He insisted that the position of the attachment (palatal) has a more important role than its design. In his study, Burashed (24) stressed that there are no differences between both types of attachments when treating an anterior open bite. Likewise, Burashed and Sebai (25) defended that there are no differences between both types of attachments when treating an overbite.

This study had a small sample size that could interfere with the correct interpretation of the statistical analysis. Another bias is the use of the movement Table of Align Technology®, which does not represent the exact movement of the tooth but gives us a range of movements. Besides, patients were treated in two different environments: a university dental clinic with postgraduate orthodontic students and a private clinic with expert orthodontics in aligner treatments.

## Conclusions

This research work yielded some conclusions regarding the choice of the correct attachment in the upper lateral incisors for rotation, angulation and inclination. Optimized attachments rotate better lateral incisors; conventional vertical attachments are more efficient to improve mesio-distal angulation; and horizontal attachments serve best for torque movements.
